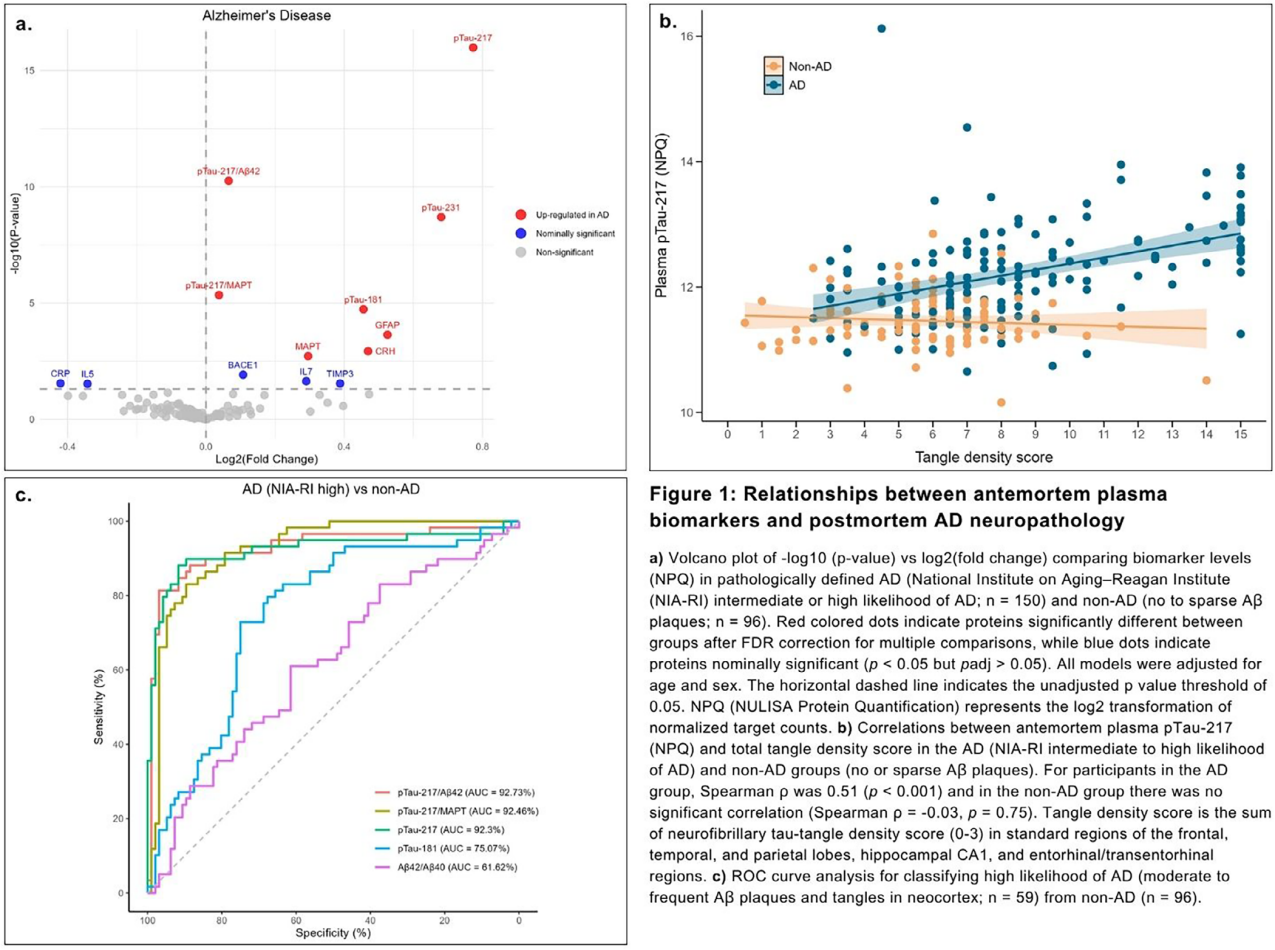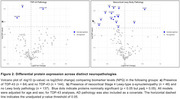# Using NULISAseq to identify biomarkers linked to postmortem Alzheimer's disease and other neurodegenerative pathologies

**DOI:** 10.1002/alz70856_099235

**Published:** 2025-12-24

**Authors:** Marisa N. Denkinger, Alpana Singh, James Liu, Kari Dieckhoff, Geidy E Serrano, Alireza Atri, Antoine Leuzy, Thomas G Beach, Eric M. Reiman, Nicholas J. Ashton

**Affiliations:** ^1^ Banner Sun Health Research Institute, Sun City, AZ, USA; ^2^ Banner Alzheimer's Institute, Phoenix, AZ, USA

## Abstract

**Background:**

Plasma biomarkers are increasingly recognized for identifying Alzheimer's disease (AD) pathology but biomarkers for other common co‐occurring pathologies (*e.g*., TDP‐43, alpha‐synuclein) are needed. Limited *in vivo* gold standards for these pathologies limit biomarker discovery to clinical assessments, which lack the specificity of neuropathological examination. To address this, we used NULISAseq to examine how plasma proteins, collected < 5‐years before death, relate to postmortem neuropathology in a cohort with diverse neurodegenerative diseases.

**Methods:**

Using the NULISAseq CNS panel, we analyzed antemortem plasma (median= 1.4 years prior to death, SD= 1.3) from 253 participants in the Brain and Body Donation Program at Banner Sun Health Research Institute. All participants underwent postmortem neuropathological examination. Linear Models for Microarray and RNA‐Seq Data (LIMMA) were used to identify differentially expressed proteins between pathology‐defined groups. We used Spearman correlations to test exploratory associations with neuropathology scores and ROC curves to evaluate plasma biomarker discriminative accuracy.

**Results:**

As expected, pTau‐217 was the most upregulated protein in AD (logFC= 0.77, *p_adj_* < 0.001; Figure 1a) and correlated with postmortem cortical tau neurofibrillary tangles in AD (ρ = 0.51, *p* < 0.001; Figure 1b) but not in non‐AD conditions (ρ= –0.03, *p* =  0.75). pTau‐217/Aβ42 had the highest accuracy in classifying AD vs non‐AD (AUC=92.73; Figure 1c). TDP‐43 pathology was associated with three proteins, including pTDP43‐409 (logFC= 0.21, *p* =  0.008; Figure 2a), but none remained significant after controlling for multiple testing. In neocortical Lewy‐type α‐synucleinopathy, sixteen proteins were differentially expressed. DDC (logFC= 0.27, *p* =  0.03; Figure 2b) and PARK7 (logFC= 0.56, *p* =  0.01) were upregulated but none remained significant after correction. Restricting samples to < 2‐years between blood draw and autopsy did not change AD findings but changed differentially expressed proteins for TDP‐43 and Lewy body pathology, which will be presented.

**Conclusion:**

Neuropathological validation is critical for establishing peripheral biomarkers of disease pathology. With limited *in vivo* gold‐standards for TDP‐43 and Lewy body pathologies, our postmortem findings show potential plasma biomarkers (*e.g*., pTDP43‐409 and DDC) that may act as proxy indicators of these pathologies. The strong association of pTau‐217 with AD neuropathology highlights the utility of NULISAseq for biomarker discovery.